# Optical coherence tomography-based misdiagnosis and morphological distinction in pachychoroid neovasculopathy vs. polypoidal choroidal vasculopathy

**DOI:** 10.1038/s41433-023-02529-5

**Published:** 2023-05-08

**Authors:** Jakob Siedlecki, Julian Klaas, Leonie Keidel, Ben Asani, Johannes Schiefelbein, Dominik Knebel, Nikolaus Luft, Siegfried G. Priglinger, Benedikt Schworm

**Affiliations:** Department of Ophthalmology, University Hospital, LMU Munich, Munich, Germany

**Keywords:** Predictive markers, Biomarkers

## Abstract

**Purpose:**

To evaluate the rate of misdiagnosis of aneurysmatic pachychoroid type 1 choroidal neovascularization/polypoidal choroidal vasculopathy (PAT1/PCV) among cases diagnosed as non-aneurysmatic pachychoroid neovasculopathy (PNV) and to define optical coherence tomography (OCT) features facilitating their distinction.

**Methods:**

The database of the Department of Ophthalmology, Ludwig-Maximilians University Munich, was screened for patients diagnosed with PNV. Multimodal imaging was screened for the presence of choroidal neovascularization (CNV) and aneurysms/polyps. Imaging features facilitating the diagnosis of PAT1/PCV were analysed.

**Results:**

In total, 49 eyes of 44 patients with a clinical PNV diagnosis were included, of which 42 (85.7%) had PNV and 7 (14.3%) represented misdiagnosed PAT1/PCV. SFCT was comparable (PNV: 377 ± 92 vs. PAT1/PCV: 400 ± 83 µm; *p* = 0.39). Whereas no difference was detected in total pigment epithelium detachment (PED) diameter (*p* = 0.46), maximum PED height was significantly higher in the PAT1/PCV group (199 ± 31 vs. 82 ± 46, *p* < 0.00001). In a receiver operating characteristic (ROC) analysis, the optimum cutoff for defining “peaking PED” was 158 µm with an area under the curve of 0.969, a sensitivity of 1.0 (95% CI: 0.59–1.0), and a specificity of 0.95 (95% CI: 0.84–0.99). Sub-retinal hyperreflective material (SHRM; p = 0.04), sub-retinal ring-like structures (SRRLS; *p* < 0.00001), and sub-RPE fluid (*p* = 0.04) were significantly more frequent in eyes with PAT1/PCV.

**Conclusion:**

A relevant percentage of eyes diagnosed with PNV might instead suffer from PAT1/PCV. The detection of a maximum PED height (“peaking PED”) exceeding approximately 150 µm, SHRM, SRRLS, and sub-RPE fluid might greatly aid in the production of a more accurate diagnosis.

## Introduction

Pachychoroid disorders of the macula represent a novel diagnostic entity characterized by pathological submacular choroidal congestion [1, 2]. In pachychoroid conditions, choroidal congestion manifests as choroidal thickening, the formation of voluminous pachyvessels in the choroid’s Haller layer, and subsequent choriocapillaris filling deficits and atrophy [1].

The spectrum of pachychoroid disorders has been recently suggested to represent a pathophysiological continuum [2]. In this continuum, non-neovascular early stages are dissimilar to later neovascular stages of disease [2]. Specifically, pachychoroid pigment epitheliopathy (PPE) as stage (i) and central serous chorioretinopathy (CSC) as stage (ii) are mainly characterized by the degeneration of retinal pigment epithelium (RPE) and subretinal fluid in the absence of neovascularization [1, 2]. Once disease progresses, neovascular complications define stages (iii) and (iv) [1, 2]. Stage (iii), namely pachychoroid neovasculopathy (PNV) first described by Pang and Freund [3], is defined as choroidal neovascularization (CNV) over areas of choroidal thickening and dilated choroidal vessels [3]. As the disease progresses further, polyps frequently build up within the CNV, defining stage (iv), namely polypoidal choroidal vasculopathy (PCV) [4]. As these “polyps” have recently been found to represent aneurysms, the alternative term of pachychoroid aneurysmal type 1 CNV (PAT1) has of late been increasingly adopted as a replacement for the term “PCV” [1, 5, 6].

Recent longitudinal data indicate that PNV can progress into PAT1/PCV [7]. On the other hand, early studies of type 1 CNV complicating CSC and the first report defining the term “PNV” suggest that many eyes present with aneurysms/polyps within the neovascular network as early as the first diagnosis [3, 8].

Whereas the diagnosis of PNV as a neovascular stage of pachychoroid disease has become markedly easier by the introduction of OCT angiography [9–11] and OCT biomarkers such as a “flat, irregular pigment epithelium detachment” [10, 12] and the “double layer sign” [13], the distinction between non-aneurysmatic PNV and aneurysmatic PAT1/PCV is noticeably more complex and may even be greatly underappreciated, given the current popularity of PNV as a novel diagnostic entity.

The aim of this study has therefore been cross-sectionally to review cases of presumed PNV for the presence of aneurysms/polyps on multimodal imaging suggesting primary misdiagnosis and to evaluate OCT-based criteria of distinction between the two disease stages within the pachychoroid spectrum.

## Methods

### Participants

For this retrospective cohort study, the smart eye database [14] of the Department of Ophthalmology, Ludwig-Maximilians University, Munich, was screened for all patients diagnosed with the keywords “pachychoroid neovasculopathy” or “PNV” in the years 2017 to 2021 as described previously [7]. Screening included all outpatient clinic patients and patients receiving anti-VEGF injections. The first visit during which PNV had been diagnosed was analysed, including standardized objective refraction corrected visual acuity testing, air-puff non-contact tonometry, slit-lamp biomicroscopy, and dilated funduscopy. Moreover, multimodal imaging was reviewed (see below). Clinical data was obtained from each patient, including age, gender, and first diagnosis of PNV.

Written informed consent was obtained at treatment initiation for the purpose of clinical management. Ethics approval for the anonymized analysis of imaging data and medical records was obtained from the ethics committee of the Ludwig-Maximilians University Munich (identifier 21–1246). The study adhered to the tenets of the Declaration of Helsinki.

### Multimodal imaging

Imaging was performed as described previously [7]. Multimodal retinal imaging (all on Spectralis HRA + OCT, Heidelberg Engineering, Heidelberg, Germany) was performed after pupil dilation with topical tropicamide 1% and phenylephrine 2.5%. It included spectral domain optical coherence tomography (SD-OCT), performed as volume scans (49 B-scans) in enhanced depth imaging (EDI) mode and near-infrared (NIR) confocal laser scanning ophthalmoscopy (CSLO), in each eye at every visit. Fluorescein (FA) and indocyanine green (ICG) angiography and additional OCT angiography scans were performed at baseline and at the discretion of the treating physician.

### Primary outcome

Rate of PAT1/PCV misdiagnosis among cases with presumed PNV.

### Secondary outcome

Differences in macular morphology on OCT between PNV and PAT1/PCV.

### Definition of the pachychoroid phenotype

The diagnosis of a pachychoroid condition was based on a multimodal approach [1]. EDI-OCT was used to assess subfoveal choroidal thickness (SFCT) and sub-lesional choroidal thickness (e.g., below pachychoroid pigment epitheliopathy or a CNV), which were interpreted as a possible pachychoroid in cases in which they showed focal or diffuse thickening above 300 µm [1]; moreover, EDI-OCT was used to assess the presence of pachyvessels (diameter of >180 µm) and an attenuation of the inner choroid in favour of a dilation in Haller’s (or Sattler’s) layer [1, 15]. In addition, dynamic angiography including FA and ICG had to show characteristic choroidal hyperpermeability or punctate hyperfluorescent spots demarcating increased extravasation of fluid and lipoprotein-bound ICGA from the choroidal lumina into the surrounding choroidal stroma [1]. In cases of doubt, OCT angiography was used to scan for choriocapillaris flow impairments (“flow deficits” or “flow voids”) [16]. Any other more likely diagnoses (e.g., inflammatory) had to be excluded on multimodal imaging.

### Definition of PAT1/PCV

As described previously [7], PAT1/PCV was defined as the presence of aneurysms/polyps within the PNV type 1 CNV network, with characteristic OCT and FA/ICGA changes. OCT signs to diagnose PAT1/PCV were defined according to the recent consensus nomenclature and non-ICGA diagnostic criteria, including sharply peaked pigment epithelium detachment, sub-retinal pigment epithelium ring-like structures, peaked/multilobular pigment epithelium detachment, double-layer sign, choroidal thickening with pachyvessels, and sub-retinal and sub-retinal pigment epithelium fluid [17]. ICGA diagnostic criteria for PAT1/PCV were applied as defined in the EVEREST study, focusing on the presence of focal hyperfluorescent lesions (=aneurysms/polyps) appearing on ICGA before Minute 6, associated with a branching vascular network/type 1 CNV [18, 19].

### Assessment of macular morphology, SFCT, exudation, and PED characteristics

In all eyes, SFCT was measured directly underneath the fovea from the outer portion of the retinal pigment epithelium to the sclerochoroidal interface. Exudation was characterized by the assessment of subretinal hyperreflective material (SHRM), subretinal fluid (SRF), intraretinal fluid (IRF), and sub-retinal pigment epithelium fluid (sub-RPEF). Moreover, PEDs were measured as described previously [20] with regard to their horizontal diameter (parallel to Bruch’s membrane) and maximum height (perpendicular to Bruch’s membrane from its inner portion to the outer portion of the RPE) and, in PAT1/PCV, both in the area of the peaking PED and in the adjacent flat irregular pigment epithelium detachment.

### Statistical analysis

All data were gathered and analysed in Microsoft Excel spreadsheets (Version 16.53 for Mac; Microsoft, Redmond, WA, USA). Statistical analysis was performed in SPSS Statistics 28 (IBM Germany GmbH, Ehningen, Germany). The Kolmogorov-Smirnov test was employed to test for normal distribution. Statistical analyses were performed using the dependent and independent two-tailed Student *t* test and the Wilcoxon signed-rank and the Mann–Whitney-U test. Fisher’s exact test was employed to compare proportions of categories between groups. A receiver operating characteristic (ROC) analysis was performed using the online tool easyROC (version 1.3.1) [21]. The Youden index was used to estimate an optimum cutoff to define “peaking PED”. The level to indicate statistical significance was defined as *p* < 0.05.

## Results

### Baseline demographics

In total, 49 eyes of 44 patients with a history of PNV diagnosis were included in the study. Of those, 42 (85.7%) had a correct diagnosis of non-aneurysmatic PNV at baseline and 7 (14.3%) represented primarily misdiagnosed aneurysmatic PAT1/PCV, in which aneurysmatic disease was not correctly recognized at first presentation. Mean age was 61.5 ± 8.2 (38.5–78.0) years. Age was comparable between the PNV and the PAT1/PCV groups (60.7 ± 8.5 (38.5–78.0) vs. 65.5 ± 5.4 (58.6–72.9) years; *p* = 0.16). The patients comprised 15 women (34.1%) and 29 men (65.9%); no difference was detected in the gender distribution between the PNV and PAT1/PCV groups (female: 35.1 vs. 28.6%; *p* > 0.99).

### Pretreatment prior to PNV / PAT1/PCV diagnosis

In the PNV group, half-fluence photodynamic therapy (PDT) had been performed for chronic CSC in 5 eyes (11.9%) a mean 2.7 ± 1.9 years prior to PNV diagnosis. Two eyes (4.8%) received nondamaging subthreshold laser treatment (Topcon Endpoint Management™, Topcon Healthcare Inc., Tokyo, Japan) for chronic CSC a mean 0.4 ± 0.5 years prior to PNV diagnosis. In the PAT1/PCV group, none of the patients had received PDT or nondamaging subthreshold laser treatment.

### SFCT and PED characteristics

**Fig. 1:**
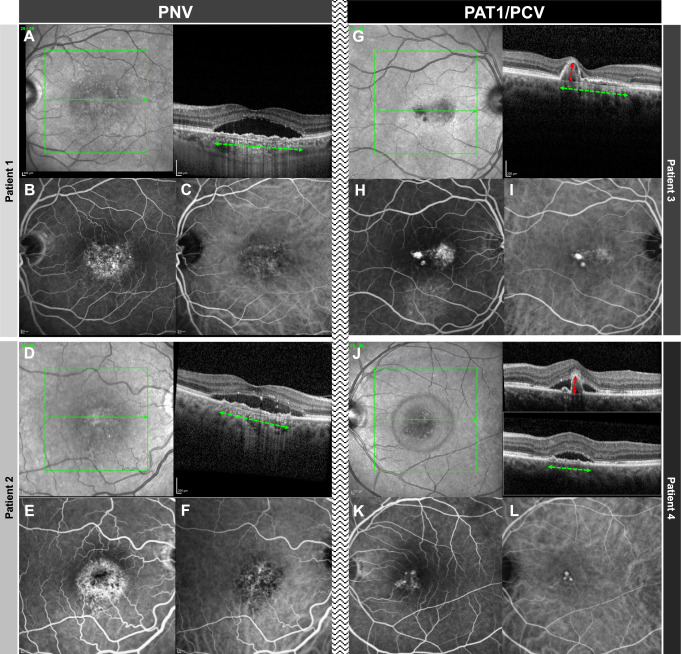
Comparison of two cases PNV and two cases of PAT1/PCV. In patients 1 and 2 with PNV, OCT (**A** + **D**) demonstrates a flat irregular PED (alternatively double layer sign) and subretinal fluid. Note that the flat irregular PED has a wide horizontal diameter (green horizontal arrow) and low height. Whereas FA (**B** + **E**) shows unspecific hyperfluorescence, ICG (**C** + **F**) shows a type 1 choroidal neovascularization without evidence of aneurysmal/polypoidal lesions. In patients 3 and 4 with PAT1/PCV, OCT (**G** + **J**) shows a peaking PED (red vertical arrow) with greater height having an adjacent double layer sign (diameter demonstrated with a green horizontal arrow). FA (**H** + **K**) shows a more focal hyperfluorescence, and ICG (**I** + **L**) clearly demonstrates the presence of aneurysms/polyps.

SFCT and PED characteristics can be found in Table 1. SFCT was comparable between the PNV and the PAT1/PCV groups (377 ± 92 (185–589) vs. 400 ± 83 (272–505) µm; *p* = 0.39). Maximum PED diameter was also similar between the groups (1809 ± 645 (934–3301) vs. 2004 ± 613 (1196–2833) µm; *p* = 0.46). Maximum PED height was significantly higher in the PAT1/PCV as compared with the PNV groups (199 ± 31 (158–245) vs. 82 ± 46 (27–267) µm; *p* < 0.00001). These “PED peaks” occupied a mean 489 ± 149 (317–707) µm horizontal diameter, representing 25 ± 8 (19–42)% of the total PED diameter in PAT1/PCV eyes. Other than the “PED peak”, PED height was comparable between both groups (PNV: 64 ± 32 (23–149) vs. PAT1/PCV: 58 ± 14 (37–72) µm; *p* = 0.60). Whereas all (100%) eyes in the PAT1/PCV group exceeded 150 µm with their peaking PED, 39 out of 42 PNV eyes (92.9%) remained below 150 µm (Fig. 1).

**Table 1:** Baseline characteristics and main outcome measures.

	PNV	PAT1/PCV	*p* value
No. of eyes	42 (85.7 %)	7 (14.3 %)	
Age (y)	60.7 ± 8.5 (38.5–78.0)	65.5 ± 5.4 (58.6–2.9)	*p* = 0.016
SFCT (µm)	377 ± 92 (185–589)	400 ± 83 (272–505)	*p* = 0.39
PED			
max. diameter (µm)	1809 ± 645 (934–3301)	2004 ± 613 (1196–2833)	*p* = 0.46
max. height (µm)	82 ± 46 (27–267)	199 ± 31 (158–245)	*p* < 0.00001
Peaking PED	0 (0 %)	7 (100 %)	*p* < 0.00001
Complex/multilobular PED	42 (100 %)	7 (100 %)	*p* > 0.99
Sub-retinal ring-like structure	0 (0 %)	5 (71.4 %)	*p* < 0.00001
SHRM	12 (28.6 %)	5 (71.4 %)	*p* = 0.04
Double-layer sign	42 (100 %)	7 (100 %)	*p* > 0.99
Fluid (actively exudating eyes)			
intraretinal	1 (2.6 %)	2 (28.6 %)	*p* = 0.056
subretinal	39 (100 %)	7 (100 %)	*p* < 0.99
sub-RPE	3 (7.7 %)	3 (42.9 %)	*p* = 0.037

Four eyes (57.1%) in the PAT1/PCV group showed one aneurysm/polyp, two (28.6%) showed two, and one eye (14.3%) showed three aneurysms/polyps on ICG angiography. Mean “peaking PED” height was 184 ± 33 (129–245) µm, and 10 out of the 11 “PED peaks” (90.9%) exceeded 150 µm.

A double layer sign was seen in all eyes in both groups (100%). A complex / multilobular PED was seen in all eyes (100%) with PAT1/PCV and in all eyes (100%) with PNV. A sub-retinal ring-like lesion was seen in 5 eyes (71.4%) with PAT1/PCV and in none of the eyes with PNV.

### Macular fluid and exudation characteristics

**Fig. 2:**
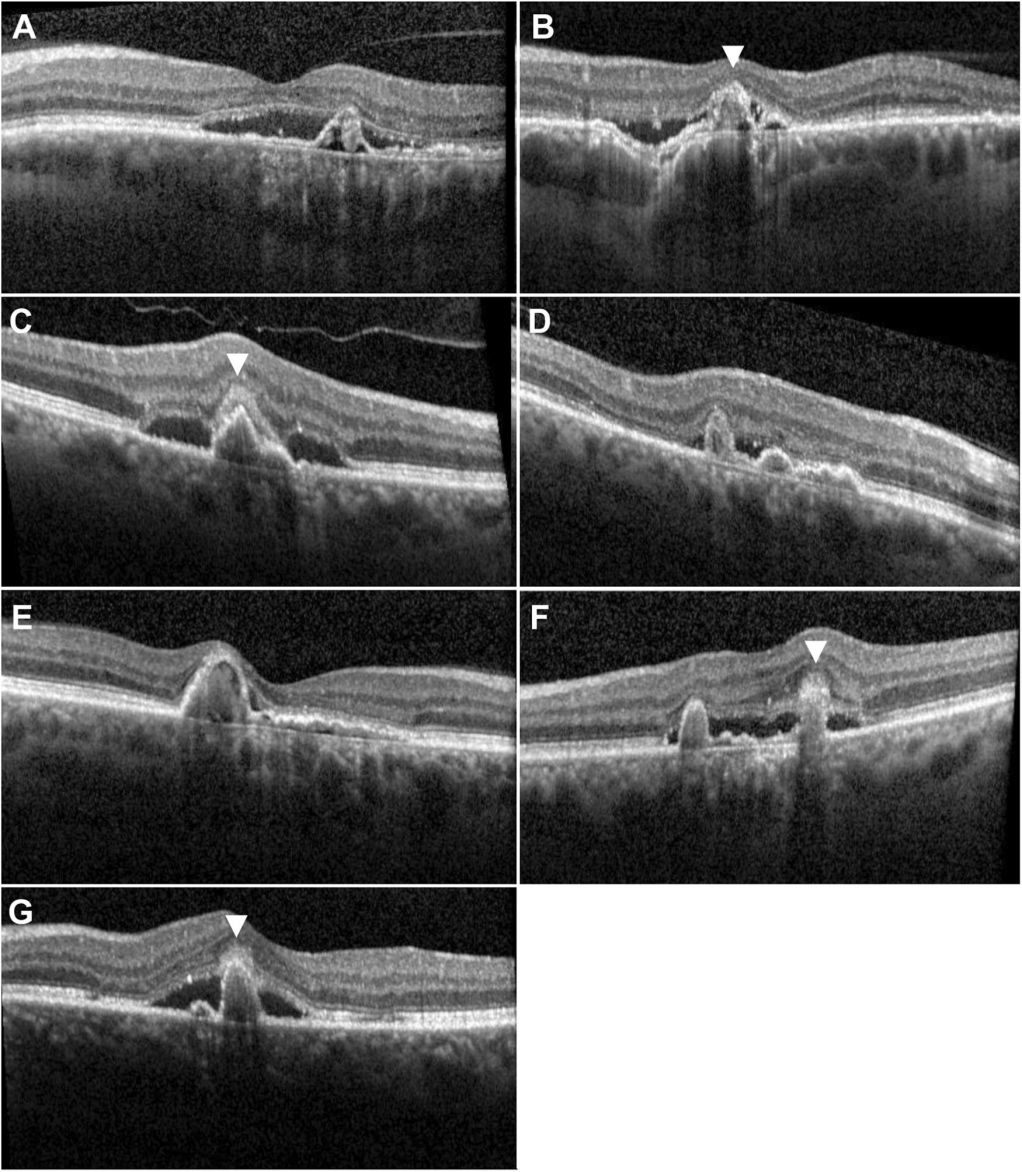
OCT scans of all seven cases of PAT1/PCV primarily misdiagnosed as PNV. All eyes (**A**–**G**) clearly show a peaking PED adjacent to a double layer sign. Note the presence of SHRM above the peaking PED in cases (**B**, **C**, **F**, and **G**). The eye in A also exhibits SHRM below the demonstrated B-scan, indicating a SHRM prevalence of 71.4 % in PAT1/PCV eyes, a value that is significantly more frequent than in PNV (28.6%, *p* = 0.04). Also note the sub-retinal ring-like structures within the peaking PED in (**A**, **B**, and **D**). All demonstrate peaking PEDs exceeded a height of 150 µm. Eyes (**A**–**D**) present with one peaking PED/aneurysm on ICG, whereas eyes (**E**), (**F**) demonstrate 2, and eye G has 3 peaking PEDs/aneurysms on OCT and ICG.

Macular fluid and exudation characteristics can be found in Table 1. In the PNV group, macular fluid was observed in 39 out of 42 eyes (92.9%). The remaining three eyes (7.1%) displayed a quiescent CNV without exudation at baseline. In the PAT1/PCV group, macular fluid was observed in all eyes (100%). SRF was seen in all actively exudating eyes (100%) in the PNV and PAT1/PCV groups (*p* > 0.99). IRF was seen in one eye in the PNV group (2.6%) and in 2 eyes in the PAT1/PCV group (28.6%; *p* = 0.056). Sub-RPEF was found in 3 eyes in each group, which was significantly more frequent in the PAT1/PCV group (42.9 vs. 7.7%, *p* = 0.037). SHRM was significantly more frequent in eyes with PAT1/PCV (71.4 vs 28.6%; *p* = 0.04) (Fig. 2). In all cases of SHRM in PAT1/PCV (100%), SHRM was found above the peak of the peaking PED. A focal choroidal excavation was found in one eye in each group (2.4 and 14.3%).

### Receiver operating characteristic (ROC) analysis

**Fig. 3:**
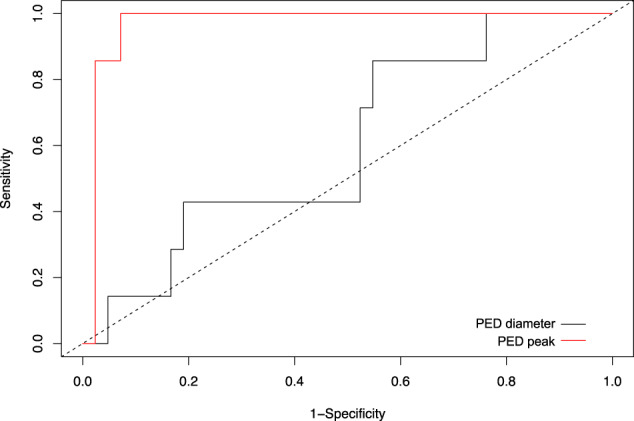
Receiver operating characteristic (ROC) analysis of PED height and diameter. The optimum cutoff used to define “peaking PED” was 158 µm with an area under the curve (AUC) of 0.969 (sensitivity 1.0 (95% confidence interval (CI): 0.59–1.0); specificity 0.93 (95 CI: 0.81–0.99)). For a PED diameter as a parameter of distinction, ROC analysis yielded markedly worse results with an AUC of 0.601 (optimum cutoff: 1598 µm); sensitivity 0.86 (95% CI: 0.42–1.0); specificity: 0.46 (95 CI: 0.31–0.63).

In a receiver operating characteristic (ROC) analysis, the optimum cutoff used to define “peaking PED” was 158 µm with an area under the curve (AUC) of 0.969 (sensitivity 1.0 (95% confidence interval (CI): 0.59–1.0); specificity 0.93 (95 CI: 0.81–0.99); Fig. 3). For PED diameter as a parameter of distinction, ROC analysis yielded markedly worse results with an AUC of 0.601 (optimum cutoff: 1598 µm); sensitivity 0.86 (95% CI: 0.42–1.0); specificity: 0.46 (95 CI: 0.31–0.63).

## Discussion

The present study indicates that a relevant percentage of eyes diagnosed with PNV in a clinical real-world setting might in reality suffer from aneurysmatic PAT1/PCV. Among 49 eyes with a clinical diagnosis of PNV in this study, we found that 7 (14.3%) showed clinical signs of aneurysmal disease on both OCT and FA/ICGA at first diagnosis but had not been correctly identified.

Whereas ICGA remains the gold standard for diagnosing PAT1/PCV, specific OCT-based diagnostic criteria for PAT1/PCV have recently been suggested [17, 22]. The establishment of such OCT criteria is important for two reasons. First, although most hospitals and private practices can readily offer OCT, FA is lacking in some, and ICGA is unavailable in many clinical institutions. Second, OCT is by far the most widespread and recognized retinal imaging method, and clinicians nowadays seem to devote most of their time to the interpretation of OCT. For these two reasons, a better definition of PAT1/PCV diagnostic criteria on OCT has become an important undertaking in order to improve clinical care [17, 22].

Pachychoroid spectrum has become a popular diagnosis, reflected in an almost exponential increase in pubmed.gov listed articles referencing this keyword, ranging from its first description by Warrow and colleagues in 2013 [23] to 44 articles in the year 2018 and 97 in 2021. However, the definitive diagnosis of pachychoroid phenotype and its distinct maculopathies might be more complex than initially thought and require far more than the single denominator of choroidal thickness [24].

In this context, our study suggests several OCT features in a real-world cohort that might greatly aid in the differentiation between PNV as a non-aneurysmatic pachychoroid related maculopathy and PAT1/PCV as an aneurysmatic pachychoroid related maculopathy. Although we found that SFCT and age did not differ between both entities, PED height, the presence of SHRM above the PED peak, sub-RPE fluid, and a SRRLS strongly suggested PAT1/PCV. These data are in good agreement with a recent study by Cheung et al. [17] who consider that the presence of a sharp-peaked PED and of SRRLS is of great diagnostic importance. Whereas we have been unable to examine the importance of a “complex RPE elevation” as mentioned in the Cheung et al study [17], because of the absence of a macular scanning pattern allowing an en-face reconstruction, our data indicate the presence of SHRM above the PED peak and the presence of sub-RPE fluid as novel additional biomarkers. At 42.9%, sub-RPE fluid was significantly more frequent in PAT1/PCV eyes than in PNV eyes (7.7%). The same applies for SHRM, which was significantly more frequent in eyes with PAT1/PCV (71.4%) than with PNV (28.6%).

Furthermore, we performed a ROC analysis to provide a value of height above which a PED could be interpreted as “peaking PED”. From our data, we found 158 µm to be an optimal cutoff for defining “peaking PED”, with an area under the curve of 0.975, a sensitivity of 1.0 (95% CI: 0.59–1.0), and a specificity of 0.95 (95 CI: 0.84–0.99). Although such a simplified approach is not intended to trivialize a differential diagnosis that sometimes represents a diagnostic conundrum, the value of 158 µm might be of use as a first indicator of PAT1/PCV, following which further imaging, especially ICG, can be recommended.

With regard to therapeutic approaches, the differentiation between PNV and PAT1/PCV harbours implications for the potential use of photodynamic therapy (PDT) with Verteporfin. Although PDT has also been described for PNV in smaller studies [25–28], high level evidence from randomized controlled trials is available for PDT in PAT1/PCV [19, 29]. The presence of polypoidal lesions particularly seems to favour the addition of PDT to intravitreal anti-VEGF therapy, as their regression seems to occur more often with combination therapy, resulting in better visual acuity outcomes [29]. Moreover, in the presence of polypoidal lesions, full-dose PDT should be preferred over reduced dose or fluence settings.

Also, differentiation between PNV and PAT1/PCV bears prognostic implications. PAT1/PCV is regarded as a more aggressive form of pachychoroid neovascularization with sometimes unpredictable exudation dynamics. Yoon et al. for example found that PAT1/PCV eyes presented more frequently with aggressive OCT biomarkers of disease like intraretinal fluid (38.2 vs. 12.2%) or macular haemorrhage (51.4 vs. 12.2%) than eyes with PNV [30]. Especially the higher incidence of macular haemorrhage in PAT1/PCV (30–63.6% [31] vs. 20.2% in PNV [32]) seems to impact long-term prognosis, with vitreous breakthrough haemorrhage often requiring pars plana vitrectomy [31]. Therefore, eyes with PAT1/PCV on average receive more annual anti-VEGF injections than eyes with PNV [30]. In this context, data from the EVEREST II trial suggest that the benefit of anti-VEGF/PDT combination therapy over anti-VEGF monotherapy mainly lies in a closure of PAT1/PCV polypoidal lesions, which was associated with higher gains in visual acuity [29] and might reduce the risk of haemorrhage.

Our study is limited by its small size and retrospective nature. Larger studies with longer follow-up are necessary, in particular to establish the definition of “peaking PED” as PED exceeding 158 µm. Concerning the rate of misdiagnosis, the cases and diagnoses included in our study span 2017 until 2021. From a 2022 perspective, recent efforts undertaken to improve the characterization of pachychoroid spectrum might already have led to clinicians distinguishing between non-aneurysmatic and aneurysmatic pachychoroid disease, thereby yielding a lower rate of misdiagnosis. A further important limitation is that our cohort exhibited primarily a pachychoroid phenotype. The OCT signs analysed in this study should therefore not be generalized to PCV in patients with an age-related macular degeneration phenotype, which presents at an older age with soft or reticular pseudodrusen and thinner choroid [33].

In conclusion, a relevant percentage of eyes clinically diagnosed with PNV might in reality suffer from aneurysmatic PAT1/PCV. In addition to the importance of ICG, which still represents the gold standard for diagnosing PAT1/PCV, our data further corroborate OCT as a suitable imaging method for differentiating between PNV and PAT1/PCV. A peaking PED exceeding 158 µm in height and the presence of SRRLS, sub-RPE fluid, and SHRM above the PED peak all suggest the presence of aneurysms and the diagnosis of PAT1/PCV instead of PNV.

## Summary

### What was known before

- Pachychoroid neovasculopathy (PNV) and polypoidal choroidal vasculopathy (PCV) both belong to the pachychoroid spectrum.
- PNV and PCV both share common imaging features.

### What this study adds

- In addition to dynamic angiography as diagnostic gold standard, optical coherence tomography (OCT) can greatly aid in differentiating both entities.
- Especially the presence of a peaking pigment epithelium detachment exceeding 150 µm height might be a strong denominator indicating PCV.

## Data Availability

Data will be made available upon reasonable request.
